# Infant Skin Barrier, Structure, and Enzymatic Activity Differ from Those of Adult in an East Asian Cohort

**DOI:** 10.1155/2018/1302465

**Published:** 2018-07-12

**Authors:** Qiwei Liu, Yanhui Zhang, Simon G. Danby, Michael J. Cork, Georgios N. Stamatas

**Affiliations:** ^1^Baby Innovation Platforms, Skin Care R&D, Johnson & Johnson Consumer China, Shanghai, China; ^2^The Academic Unit of Dermatology Research, Department of Infection and Immunity, Faculty of Medicine, Dentistry and Health, The University of Sheffield Medical School, Beech Hill Road, Sheffield, UK; ^3^Baby Innovation Platforms, Skin Care R&D, Johnson & Johnson Santé Beauté France, Issy-les-Moulineaux, France

## Abstract

Skin physiology is dynamically changing over the first years of postnatal life; however, ethnic variations are still unclear. The aim of this study was to characterize infant skin barrier function, epidermal structure, and desquamation-related enzymatic activity as compared to that of adult skin in an East Asian population. The skin properties of 52 infants (3-24 months) and 27 adults (20-40 years) were assessed by noninvasive methods at the dorsal forearm and upper inner arm. Transepidermal water loss and skin surface conductance values were higher and more dispersed for infants compared to adults. Infant skin surface pH was slightly lower than adult on the dorsal forearm. The infant SC and viable epidermis were thinner compared to adults with differences that were site-specific. Although the chymotrypsin-like activity for infant skin was comparable to adult level, the caseinolytic specific activity was significantly higher for the infant cohort. These observations indicate a differently controlled pattern of corneocyte desquamation in infants. In conclusion, structural and functional differences exist between infant and adult skin in the East Asian population pointing to dynamic maturation of the epidermal barrier early in life.

## 1. Introduction

As the outermost organ of the body, skin is endowed with multiple functions such as protection, secretion, absorption, and thermoregulation [1]. These functions are essential throughout life. Several studies emphasize the importance of the stratum corneum (SC) in providing a protective barrier from an early age [2, 3]. At birth, the skin of term newborns behaves as a competent physical inside-out and outside-in barrier, but not at the capacity of adult skin [4–6]. The barrier function and the water-handling properties of infant SC have been shown to be different from those of adult [7, 8]. Infant SC was found to have higher water content and higher rates of transepidermal water loss (TEWL) at rest, absorb more water, and lose excess water faster than adult SC. Further studies revealed that such functional differences were associated with differences in the epidermal microstructure [9, 10].

An important factor for a healthy epidermal barrier is a carefully controlled SC desquamation process [11, 12]. SC desquamation is a proteinase-dependent process that involves complex synergies of enzymatic activities, which are responsible for precisely degrading a variety of intracellular and intercellular macromolecules and for providing an inconspicuous shedding of corneocytes [13, 14]. The corneodesmosomes are critical for the maintenance of the SC integrity and barrier function. A key event that eventually results in normal desquamation is the complete proteolysis of all corneodesmosomes.

Four distinct types of proteolytic activity are identified within the intercellular regions of the SC that are responsible for corneocyte dissociation and desquamation processes [13, 14]: (i) chymotrypsin-like protease activity exhibited by kallikrein (KLK) 7, previously referred to as stratum corneum chymotryptic enzyme (SCCE); (ii) trypsin–like protease activity exhibited by a range of kallikreins expressed in the SC, but largely in terms of overall activity accounted for by KLK5, previously stratum corneum tryptic enzyme (SCTE) and KLK14; (iii) cysteine protease activity exhibited by cathepsin L2, previously stratum corneum thiol protease (SCTP) and cathepsin L-like protease; and (iv) the aspartic protease cathepsin D. Among them, the kallikreins are the most widely studied. They play a major role in desquamation by cleaving the extracellular components of the corneodesmosomes. While the cathepsins are most active at low pH, characteristic of the skin surface [15], kallikreins exhibit only residual activity at this low pH. However, when skin surface pH increases, for example, as a result of inflammation or washing with a harsh soap, the activity of kallikreins elevates substantially, suggesting that they play an important role in facilitating skin barrier breakdown under such conditions.

To our knowledge, the involvement of desquamation and the underlying enzymatic processes in skin maturation at an early age have not been studied thus far. Given the higher keratinocyte turnover rates and the thinner epidermal layers in infant skin compared to adult [9], a closer look at parameters, such as desquamation-related enzyme activities, will benefit our holistic understanding of skin barrier maturation. Furthermore, although some fundamental studies have evaluated the structural and functional differences in skin barrier properties that have been linked with age, regional, seasonal, and ethnic background [16, 17], little is known about skin physiology and maturation in East Asian infants [8, 18], whose skin may differ from that of Caucasians in some respects [19].

In the present study, we attempt to investigate the skin water-handling properties, the skin surface pH, the skin microstructure, and the biochemical events involved in desquamation in a cohort of East Asian infants and how these properties relate to the corresponding ones of adult skin in this population.

## 2. Materials and Methods

### 2.1. Clinical Protocol

An observational, noninterventional, prospective study was conducted in the Shanghai area during the months between January and April. Fair complexioned infants of Chinese descent (both parents) and their mothers were recruited according to the inclusion and exclusion criteria shown in Table 1. The study was performed under the approval from an independent institutional review board and following the principles of the Declaration of Helsinki and the Good Clinical Practice Guideline. Adult subjects (mothers) signed a written informed consent for their participating infants and themselves (when applicable). Parents were instructed to avoid use of skin care products for themselves and the infants on the arms and the legs, for at least 24 hours before the measurements. Measurements on the arms were taken after subjects acclimated to an environmentally controlled room (20–25°C and 40-60% relative humidity) for a minimum of 15 min. Visibly distressed or crying infants were excluded. Measurements were performed on the upper inner arm (relatively protected), the dorsal forearm (relatively exposed), or the lower thigh area as described below.

### 2.2. Measurements of Skin Barrier Function, Skin Surface Hydration and Skin pH

Measurements of skin barrier function, skin surface hydration, and skin pH were performed on the dorsal forearm and the upper inner arm. The quality of the skin barrier function was assessed by measuring the TEWL rates using a closed chamber device, VapoMeter (Delfin, Kuopio, Finland). Skin surface hydration levels were assessed by measuring the electrical conductance of the skin using Skicon (IBS Ltd., Hamamatsu, Japan). This device measures the high frequency skin conductance, which is related to the water content of the superficial SC layers. Skin surface pH was measured using a Skin–pH–Meter pH 905 (Courage & Khazaka, Cologne, Germany). This instrument includes a flat glass electrode probe containing all the sensor elements, connected to a probe handle containing the measurement electronics.

### 2.3. Measurement of Skin Structure

Skin sites of interest on the lower thigh and the upper inner arm were imaged using an* in vivo* reflectance CLSM (Vivascope 1500, Lucid Inc., Henrietta, New York, USA) equipped with a laser at 830 nm (laser power < 25 mWatt at the tissue surface). This microscope generates a series of consecutive optical sections every 1 *μ*m at increasing depths. Imaging starts at the top layer of the SC and progresses down through the epidermal layers, the dermal epidermal junction, and the top layers of the dermis. The SC thickness was calculated following the method described elsewhere [9]. Briefly, SC thickness is the product of the number of images from the first image (top corneocyte layer) to the image just before the one where granular cells can be detected, times the imaging step (1 *μ*m). Similarly, the thickness of the suprapapillary epidermis (SPE) was calculated as the number of images from the top corneocyte layer to the image just before the one where the top of the dermal papilla can be detected, times the imaging step [9]. Three replicate stacks within a 4 x 4 mm^2^ area were sampled.

### 2.4. Protease Activity Assays

The SC surface was sampled using D-Squame tapes (CuDerm, Dallas, Texas, USA), applied on the left or the right ventral forearm randomly, and then immediately stored in a –20°C refrigerated container for later analysis of protease activity. Assessment of protease activity was made on samples comprising three consecutive D-Squame discs as previously described [20]. Caseinolytic and chymotrypsin-like activities were determined using EnzCheck® (Life Technologies Ltd., Paisley, UK) and MeOSuc-Arg-Pro-Tyr-AMC (Peptide Protein Research Ltd, Funtley, UK) substrates respectively.

### 2.5. Data Analysis

All statistical tests were executed using Graphpad Prism v6.0b (Graphpad Software Inc., La Jolla, California, USA) and SPSS Statistics v17 (SPSS Inc., Chicago, Illinois, USA). All data are presented as average ± one standard deviation. Comparisons between infant and adult data groups were performed using Student's* t*-test following confirmation of the normality of the distributions. Statistical significance for the difference between the two groups was accepted at the level of* p*<0.050. Data are presented as box-and-whiskers graph following Tukey's method as coded in the Graphpad software.

## 3. Results

Of the recruited subjects 52 infants and 27 adults followed the inclusion criteria. The infants were evenly spread between genders (25 males and 27 females). Their ages ranged from 3 to 24 months (mean: 12.15, median: 11.5, standard deviation: 5.67). The adults were all female (mothers) with an age range of 20-40 years (mean: 31, median: 31, standard deviation: 4.24).

### 3.1. Infant Skin Barrier Properties Differ Compared to Adult

**Figure 1:**
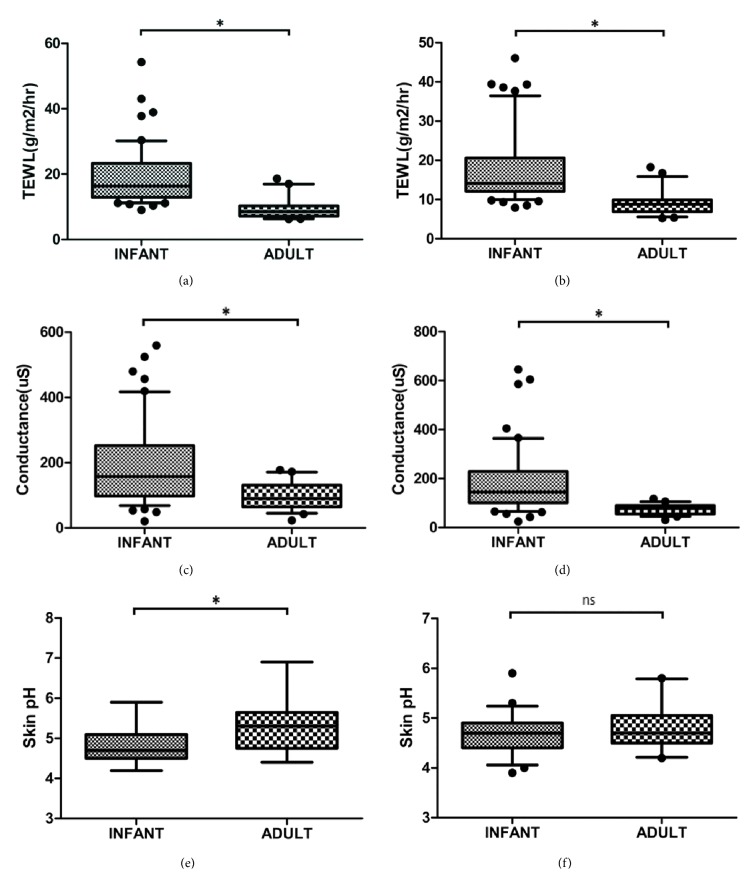
Infant skin barrier properties are different compared to those of adult skin in an East Asian population. Transepidermal water loss rates were significantly higher for infants compared to adults on (a) the upper inner arm site and (b) the dorsal forearm site. Skin conductance was significantly higher for infants compared to adults on (c) the upper inner arm site (p<0.001) and (d) the dorsal forearm site. Skin surface pH values were significantly lower for infants compared to adults on (e) the upper inner arm, but not statistically different on (f) dorsal forearm site. *∗* indicates p < 0.050.

The measured parameters relating to skin barrier and the comparison between infant and adult skin are shown in Figure 1. TEWL rates were significantly higher for infants compared to adults for both the upper inner arm (Figure 1(a)) and the dorsal arm site (Figure 1(b)). Moreover, infant skin surface hydration was elevated compared to adults as it was evidenced by the higher skin conductance values for both skin sites tested (Figures 1(c) and 1(d)).

The average skin surface pH value was slightly but statistically lower in infants (pH 4.8±0.4) than that in adults (pH 5.3±0.5) for the upper inner arm, but not for the dorsal arm site where there was no significant difference (Figures 1(e) and 1(f)).

### 3.2. Infant Epidermal Microstructure Is Different from That of Adult

**Figure 2:**
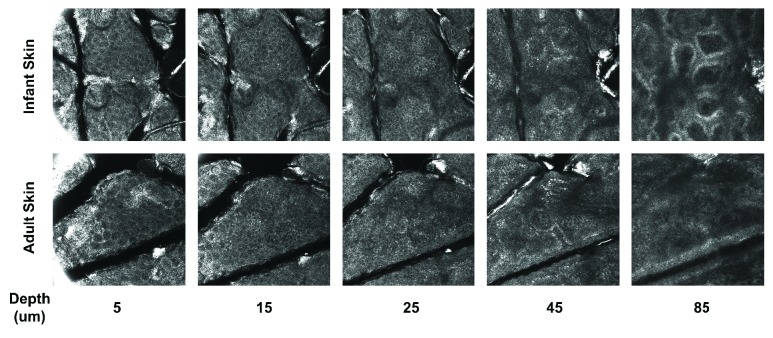
*In vivo* confocal laser scanning microscopy revealed structural differences between adult and infant skin beneath the skin surface. Representative images are shown acquired on the thigh area and at different depths representing different epidermal layers. The images at 5 *μ*m show the layer where the first granular cells appear, immediately below the brightly scattering SC. Infant skin appeared to possess denser microrelief lines close to the skin surface (images at 5 and 10 *μ*m) and denser and more uniform dermal papillae (observed at depths 45 and 85 *μ*m) compared to adult skin.

*In vivo* CLSM images revealed structural differences between infant and adult epidermis (Figure 2). Infant skin surface appeared to have denser microrelief lines and smaller SC island structures in-between these lines compared to adult skin. The dermal papillae displayed a quite homogeneous distribution in infant skin, whereas in adults they were fewer in density and more irregular in shape.

**Figure 3:**
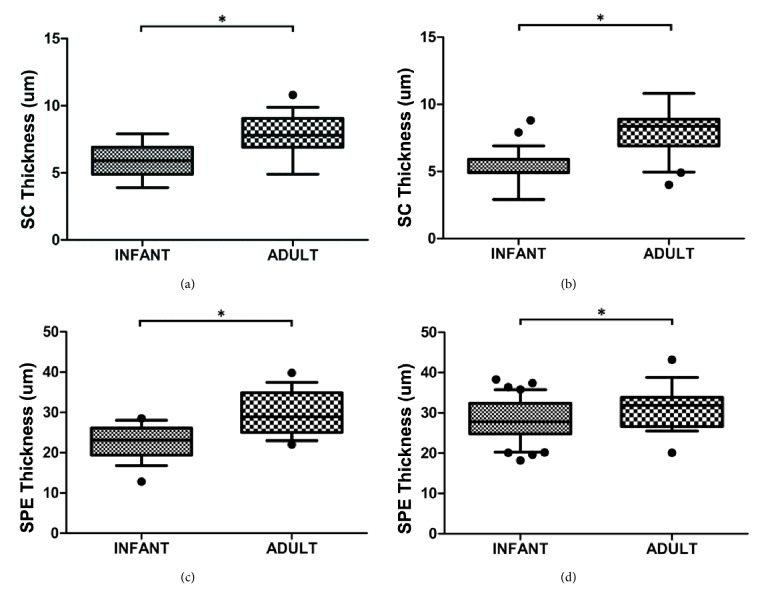
The stratum corneum (SC) and suprapapillary epidermis (SPE) were significantly thinner in infant skin compared to adult skin as measured by* in vivo* confocal laser scanning microscopy. (a) SC thickness at the upper inner arm site; (b) SC thickness at the thigh area; (c) SPE thickness at the upper inner arm site; (d) SPE thickness at the thigh area. *∗* indicates p < 0.050.

Infant SC and SPE were thinner than in adults (Figure 3). On the upper inner arm, the infant SC was on average 18% thinner than that of adults, whereas the infant SPE was on average 22% thinner than that of adults. On the thigh, the differences were 34% and 8% correspondingly for the SC and the SPE thickness.

### 3.3. Infant Skin Surface Shows Higher Caseinolytic Specific Activity Compared to Adult

**Figure 4:**
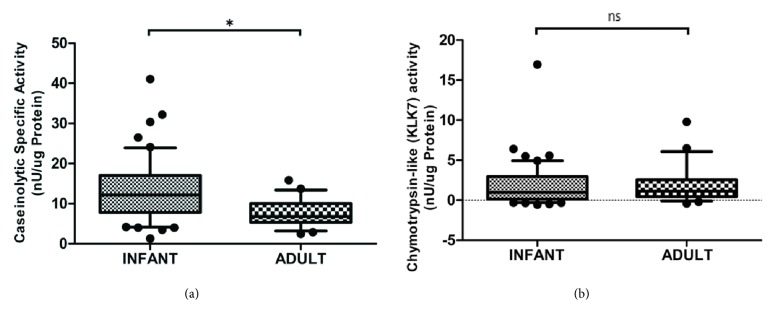
The skin surface enzymatic activity in infants differs from that of adults. (a) The caseinolytic specific activity is higher on infant compared to adult skin. (b) The chymotrypsin-like activity shows no significant difference between infant and adult skin. *∗* indicates p < 0.050 and ns indicates p > 0.050.

Significantly elevated caseinolytic specific activity was observed in infant SC samples (13.1±8.0 *μ*U/*μ*g Protein) compared to adult (7.6±3.5 *μ*U/*μ*g Protein), while no significant difference in chymotrypsin-like activity was found between infant and adult skin (Figure 4).

There are no statistical differences between male and female infants for all measured parameters.

## 4. Discussion

The skin barrier function depends on several complex and interdependent processes. Corneocyte hydration, skin surface acidification, and enzymatic corneodesmolysis are networked in a dynamic interrelationship to influence barrier homeostasis. During the first years of life infant skin undergoes a maturation process of its barrier function, which involves the careful interplay of such processes. The goal of the present study was to compare the infant skin barrier and the underlying processes with that of adult skin in a population of East Asian infants. The results demonstrated that, in this population measurable differences between infant and adult skin, comparable to those previously published for other ethnic and geographic populations. Moreover, this study was to our knowledge the first to demonstrate that desquamatory processes are involved during the period of skin maturation.

Previous studies have demonstrated that in Caucasian populations infant skin physiology differs to that of adults with respect to its structure, function, and composition [7, 9, 21]. In this study, the values of multiple barrier-relating parameters of infants were compared to those of adults in an East Asian population. Evaluations of the SC barrier were performed by common noninvasive measurements like TEWL, skin conductance, and skin surface pH. The results are in good accordance with previously published data for infants [22] and adults [23]. Infant skin was found to exhibit higher TEWL rates indicating that infant skin barrier undergoes a maturation process during first years of life [6–8, 24]. In agreement with previous findings [7, 8], these data also demonstrated higher skin surface conductance values for infant skin compared to adults [22, 23].

Besides the water-handling properties, skin structure differed between the two groups. Infant SC and SPE were found to be thinner than adult, indicating the continuous structural maturation of the infant skin. Our results showed that the SC on the thigh was on average 5.3±1.4 *μ*m and 7.9±1.8 *μ*m in thickness, respectively, for East Asian infants and adults, compared to the previously reported SC thickness of 7.3±1.1 *μ*m and 10.5±2.1 *μ*m for their Caucasian counterparts [9]. The finding that the SC was about 20-30% thinner in this East Asian population compared to Caucasian skin provides* in vivo* experimental evidence confirmed the hypothesis that Asian SC may be thinner, since the number of tape strips required to cause a determined increase in TEWL values was less [25]. Moreover, the incidence of atopic dermatitis, a condition clearly relating to the skin barrier, has been reported to be increased for East Asian compared to Caucasian children [26]. East Asian skin appears to be more sensitive to irritants and other negative environmental factors, such as pollution or weather extremes [8], possibly due to a thinner SC.

Corneocyte desquamation is a proteinase-dependent process that involves complex orchestration of enzymatic activities, which are responsible for precisely degrading a variety of intracellular and intercellular macromolecules to provide an inconspicuous shedding of single corneocytes. It is a precisely controlled cascade of events, the molecular mechanisms of which are only known in part. The role of skin surface pH in SC cohesion and desquamation through modulations of specific enzymatic activity has been demonstrated using super-bases topically applied on hairless mouse skin [27]. Immediately after birth, skin surface pH has been reported to be higher in newborns compared to adults [6]. Such increases in SC pH lead to elevated activities of serine proteases, such as KLK5, KLK7, and KLK14 [12, 28], which in turn result in modified desquamation. It is widely recognized that the surface pH of human adult skin is acidic with pH values in the range between 5 and 5.5 [29, 30]. The results reported in this study show that for infants older than 3 months the skin surface pH decreases compared to the reported values for newborn infants [6] and on the upper inner arm site remains at a slightly more acidic level compared to adults. This slight acidification may reflect a higher mobility of the H^+^ ions in a SC with higher water content (as evidenced by the higher skin surface conductance values).

In agreement with the acidic properties of the SC, the chymotrypsin-like activity, associated with the serine protease KLK7, was similar in infants and adults. Broad protease activity was elevated however, indicating that other proteases, perhaps cathepsins with acidic pH optima, are more active [31]. The cathepsins are capable of cleaving the extracellular components of corneodesmosomes and therefore facilitate desquamation. An increased rate of desquamation helps explain why infant skin is thinner compared to adults. In addition to regulating desquamation, pH regulates also the processing of lipid precursors into the components of the lipid lamellae, a highly ordered lipid matrix that contributes to the permeability barrier function of the SC. The slightly more acidic pH of infant skin compared to adult skin on the upper inner arm will support the formation of lipid lamellae, which has been shown to develop following birth [32].

One limitation of this study is that we used only female adult subjects, the children's mothers, whereas the infant subjects were equally distributed between the two genders. However, analysis showed that there are no statistical differences between male and female infants for all measured parameters, which warrants the grouping all infants in a single group.

## 5. Conclusion

The results presented in this study confirm previous observations with regard to infant skin maturation as well as provide new insights into these processes. This study confirmed that there are functional and structural differences between infant and adult skin. Parameters such as SC water content, water permeability, and skin surface pH are undergoing a maturation process. Increased water permeability may be at least partly explained by the thinner SC in infants, which is reported for the first time in this study for East Asian skin. In turn, differences in SC thickness may be explained by the newly presented data on the higher activity of certain proteolytic enzymes that confer a more complete degradation of the corneodesmosomes in infant skin. The way SC stores and transports water and the mode for normal SC desquamation and metabolic activities take years to fully reach adult levels [8]. Taken together, the presented data may help explain the increased incidence of atopic dermatitis in children of East Asian origin [26].

## Figures and Tables

**Table 1:** Inclusion and exclusion criteria for study enrollment.

**A. Inclusion Criteria**	
1. Healthy, male or female infants aged 3 – 24 months and their mothers.	
2. The mother subject must be 25-46 years of age and must be willing to present proof of guardianship.	
3. For both groups mother and child must be generally in good health as determined from a medical history. Both mother and baby should be without any family history of atopic dermatitis.	
4. The participating mother is the one who regularly cares for the child. The mother must be willing and able to follow all study directions, accept skin examination during the study.	
5. The mother must agree for her child and herself not to participate in another study during this study.	
**B. Exclusion criteria**	
1. Subjects with known allergy, particularly to adhesive tapes or latex.	
2. Subjects with excessively dry, red, or irritated skin on any part of the body.	
3. Subjects with obvious skin conditions on any part of the body that could interfere with the outcome of the study, including psoriasis, eczema, rashes, broken skin, sunburn, hyperhidrosis etc.	
4. Subjects who have fever or a known viral or bacterial infection at the beginning of the study.	
5. Mother subjects pregnant at the beginning of study.	
6. Subjects participating in any other clinical study.	

## Data Availability

The data are available upon request from the authors.
